# Acute Responses of Youth Elite Players to a Football Match in Terms of Blood Markers

**DOI:** 10.3390/sports11120242

**Published:** 2023-12-11

**Authors:** André Montanholi Fornaziero, Luiz Fernando Novack, Vitor Bertoli Nascimento, Raul Osiecki

**Affiliations:** Department of Physical Education, Federal University of Parana (UFPR), Curitiba 81531-980, Brazil; lfnovack@hotmail.com (L.F.N.); vitor.nascimento@ufpr.br (V.B.N.); raulfisioex@gmail.com (R.O.)

**Keywords:** football players, match analysis, leukocytes, cortisol, creatine kinase, lactate dehydrogenase

## Abstract

The current study verified the acute responses of participants to a football match in terms of blood markers. Sixteen elite U-18 male football players were divided into two groups: experimental (EG, *n* = 10), who played a friendly football match; and control (CG), who were not exposed to any physical exertion. Intravenous blood samples were collected from both groups at baseline, pre-match, half-time, and post-match. The blood analysis consisted of four groups: immunological (leukocytes, platelets, and cortisol), muscle damage (creatine kinase and lactate dehydrogenase), metabolic (lactate, glucose, erythrocytes, hematocrit, hemoglobin, and urea), and electrolytic (sodium, calcium, and potassium). Edwards’ training impulse demonstrated that the first half was more demanding than the second half (*p* = 0.020). Significant changes between time points and groups were observed for leukocytes (pre-match: 6920 ± 1949; post-match: 13,890 ± 3292; *p* ≤ 0.05) and cortisol (pre-match: 10.78 ± 3.63; post-match: 19.15 ± 7.40; *p* ≤ 0.05). CK (pre-match: 516.50 ± 248.38; post-match: 713.70 ± 308.20; *p* ≤ 0.05) and LDH (pre-match: 348.80 ± 36.49; post-match: 414.80 ± 26.55; *p* ≤ 0.05) increased significantly across the time points for the EG, with no difference between the groups, however. Raised lactate (pre-match: 1.05 ± 0.32; post-match: 3.24 ± 1.60; *p* ≤ 0.05) and glucose (pre-match: 72.54 ± 9.76; post-match: 101.42 ± 19.87; *p* ≤ 0.05) differences between the groups at half-time were also observed. These current findings provide helpful information to better understand football match demands regarding physiological effects.

## 1. Introduction

Football is a sport characterized by high-intensity physical actions such as sprints, jumps, and accelerations, interspersed with low-intensity actions such as jogging, walking, and even passive breaks [1,2,3,4]. These demands can be demonstrated by the total distances covered, which can vary between 9 and 13 km, and, typically, midfielders present the highest values [1,5,6,7,8]. Concerning high-intensity requirements, athletes perform between 150 and 250 actions between 15 and 20 m, representing about 30% of the total match duration [9]. In addition, the total distance covered by players in sprints usually comprises between 5 and 10% of the total distance covered [9,10], with a frequency of one action every 90 s [9].

The performance of all these high-intensity efforts requires physiological responses, which can be expressed through multiple indicators, concerning heart rate monitoring (HRM) and blood markers. HRM is considered the most used method to assess exercise intensity in the field [11]. During football matches, athletes spend approximately 65% of the total time at intensities between 70 and 90% of HRmax, and this figure is rarely below 65% HRmax [1]. Additionally, the training impulse calculation (TRIMP), proposed by Banister [12] and modified by several other researchers, is broadly used to determine the internal load demands [12,13,14]. There are several ways to calculate the TRIMP; however, the main concept is multiplying the duration of the session by its overall intensity or breaking it down into intensity zones [15].

Regarding blood markers, a multitude of enzymes, hormones, and other substances may also be used to assess the magnitude of the physiological changes, as they are highly affected in response to the metabolic disturbances caused by physical activity. One of these blood markers is creatine kinase (CK), which is an enzyme found in the skeletal muscle, heart muscle, and brain and plays an important role in adenosine diphosphate (ADP)’s conversion to adenosine triphosphate (ATP) [16]. Likewise, lactate dehydrogenase (LDH) is an enzyme present in almost all body tissue and is responsible for the conversion of lactate to pyruvate during anaerobic metabolism [16]. Both enzymes are widely assessed in sports as indicators of muscle damage; however, despite demonstrating an immediate effect following exercise, these markers are more sensitive to longer-term responses [17,18,19,20,21,22,23,24,25,26,27].

Muscle-related disturbances as an outcome of physical effort consequently increase the body’s defense system, which can be evidenced by several blood markers such as white cell count (leukogram), platelet, and cortisol analysis [17,18,19]. It is widely known that exertion brings changes in the immune system, increasing the number of circulating leukocytes which, in turn, are responsible for framing inflammatory and cellular responses to injury, infectious disease, or pathogens [16,28]. Likewise, platelets play a key role in the immune system as they are involved in the primary process of homeostasis and coagulation [29]. Cortisol is a steroid hormone that is involved in numerous functions, mainly mediation of the stress response and inflammatory response, immune function, and metabolism regulation [30]. In addition, it can also be related to higher muscle protein degradation in response to the inflammatory process caused by muscle micro-injuries [30].

Physical effort can also be analyzed using metabolic blood markers, such as glucose and lactate, which are usually closely linked to exercise intensity [31]. Blood glucose analysis might provide useful information on whether the glycogen mobilization level is coping with the energetic demands. Similarly, blood lactate concentration is commonly assessed to quantify the physiological response to exertion [32] and can be an important indicator of anaerobic and aerobic metabolism [20]. Furthermore, considerable loss of fluids might occur following a match, possibly leading to dehydration status; therefore, the analysis of blood electrolyte concentrations such as sodium, calcium, and potassium can also be useful in this scenario [16].

Despite some researchers having already analyzed the physiological responses that occur following a football match, including a control group (CG) in the methodological design in association with half-time analysis has been scarcely considered. Therefore, the present study aimed to compare the acute immunological, muscle damage, metabolic, and electrolytic blood marker responses following the completion of a football match, when comparing players who played with players who had not taken part in the match. We hypothesized that the match would increase immunological, muscle damage, and metabolic blood markers and decrease the electrolytic ones, with no changes in the CG.

## 2. Materials and Methods

### 2.1. Participants

Sixteen youth male elite football players from a second-division Brazilian league (excluding goalkeepers; age: 18.2 ± 0.5 years old; height: 179.3 ± 6.4 cm; body weight: 77.4 ± 5.0 kg; %bodyfat: 11.5 ± 1.2%; VO2max: 56.6 ± 5.5 mL/kg/min). The subjects were full-time professional athletes, with at least six years of experience in high-performance football training and competitions. The typical weekly schedule involved six training sessions from Monday to Friday, whereas on Tuesdays, double sessions were performed. The sessions lasted between 1.30 h and 2.30 h and comprised mixed football-related contents such as small-sided games, large-sided games, possession games, set-pieces, and strength/power tasks. On the weekend, the players took part in official matches for their age group, with them usually taking place on Saturdays. The experimental protocol was performed during the in-season phase.

The participants were healthy, with no signs of illness, injury, or even acute fatigue from previous training sessions. To be included in the present study, the participants had to fully complete six training sessions prior to the match without any signs of physical restriction. An exclusion criterion was established in case any player sustained any kind of injury or illness that could minimally affect their performance. Any kind of medicine intake was forbidden on the experimental day. The research was performed under the ethical standards of the Helsinki Declaration and written informed consent forms were obtained from all subjects before the start of the experiment. The study was approved by the Federal University of Parana Ethics Committee (0081.0.091.000-08).

The sample size was calculated using G*Power software (version 3.1.9.4, University of Dusseldorf, Dusseldorf, Germany) considering the following parameters for the F-test: an effect size of 0.35 (partial-eta-squared, ηp^2^), a power of 0.80, an α error of 0.05 (*p* ≤ 0.05), correlation among repeated measurements of 0.5, and a nonsphericity correction of 1. A total sample size of 14 individuals was specified.

### 2.2. Procedures

A week prior to the experimental protocol, anthropometric measurements were taken, according to the protocol of Heyward and Stolarczyk (1996) [33]. The participants were assessed during the morning and wore light football training clothing without socks. Standing height (cm) was measured with a precision of 0.1 cm using a stadiometer (Sanny Standard ES2030, Sao Paulo, SP, Brazil); the players were asked to take a deep breath and stand as tall as possible. Body weight (kg) was recorded with a digital scale (Toledo 2096PP, Sao Bernardo do Campo, SP, Brazil) to the nearest 0.1 kg. Skinfolds (subscapular, triceps, supra-iliac, and abdominal) were assessed using a caliper with 0.1 mm precision (Cescorf Scientific, Porto Alegre, RS, Brazil), and the percentage of body fat was estimated using the equation proposed by Faulkner (1968) [34].

Subsequently, each participant completed a maximal-effort progressive test on a treadmill (X-Fit 7 Power, Koropi, Greece) in order for their VO2max and HRmax to be obtained. During the test, the inclination was maintained at 1% while the speed started at 8 km/h and increased 1 km/h every 1 min until volitional fatigue. The participants’ heart rate was recorded every 15 s (Polar Electro Oy T-31 strap, Kempele, Finland), and their HRmax was defined as the highest value observed during the test. For VO2max identification, gas exchange was analyzed with a metabolic cart (Parvo Medics TrueOne 2400 metabolic cart, Salt Lake City, UT, USA). The test was considered maximal if at least two of the following criteria were met: a respiratory exchange ratio ≥ 1.15; a plateau in oxygen consumption with increasing workload; HR measured ± 10 bpm vs. predicted (220-age); or the presence of apparent signs of maximal effort (hyperpnea, facial flushing, and ataxic gait) [35,36]. Table 1 presents the comparison of the anthropometric and cardiorespiratory characteristics between the EG and CG.

The subjects played a 90 min (2 halves of 45 min) friendly match against a similar competitive-level opponent following FIFA rules, but no substitutions were allowed. National-level accredited referees worked during the experiment in order to ensure all official football rules were respected. The match was played at 3:00 p.m. (local time) on a natural grass pitch with 105 × 68 m dimensions. Eight ball boys were positioned around the pitch to quickly restart the game whenever the ball was out of play.

All players belonged to the same team and were divided into two groups: an experimental group (EG) with ten starter players and a control group (CG) with six non-starters. The EG performed the entire game without any substitution, whereas the CG was not exposed to any physical exertion before and during the match. The criteria applied for the players’ group allocation was purely football-related, and they were applied only by the club’s technical staff as it was part of a typical training week. The subjects were informed about their allocation right before the warm-up stage.

Four blood samples were collected from each player (EG and CG): (1) baseline: after 12 h fasting (8 a.m.); (2) pre-match: before the warm-up stage (2:30 p.m.); (3) half-time: immediately after finishing the first half (3:45 p.m.); and (4) post-match: immediately after finishing the second half (4:45 p.m.). Ten milliliters of blood samples were obtained and stored in proper tubes at a temperature range between −10 and −15 °C until analyzed at the laboratory.

On the experimental day, the athletes followed their typical dietary pattern with regard to breakfast and lunch intake, and during the match, only water was offered ad libitum. Eritogram, hematocrit, hemoglobin, platelets, and leukocytes were analyzed using an electrical impedance system (Abbott Cell-Dynn 3000, Chicago, IL, USA), whereas CK, LDH, urea, glucose, and calcium were determined by the enzymatic colorimetric method (Biotecnica Targa BT-3000, Rome, Italy). The selective ion technique (Drake Iselab, Sao Jose do Rio Preto, SP, Brazil) was applied to analyze sodium and potassium, and for cortisol, electrochemiluminescence (Roche Elecsys 2010, Basel, Switzerland) was utilized.

Throughout the whole match, the players used the Polar Team System heart rate transmitter (Polar Electro Oy, Kempele, Finland), which stores data every 5 s. Training impulse (TRIMP) was calculated based on the HR data following the technique proposed by Edwards (1993) [13]. According to the main institution of weather monitoring in the state of Parana/Brazil (SIMEPAR), the temperature and humidity at the start of the match were 28.1 °C and 51.6%, respectively. Food was not available between the pre-match and post-match stages, and water was offered ad libitum before the warm-up stage (after pre-match blood collection) and at half-time. Water intake was registered for each player on both occasions (Table 2).

### 2.3. Statistical Analysis

The data are presented as the mean and standard deviation (SD), and data normality was confirmed by the Kolmogorov–Smirnoff test. A one-way analysis of variance (ANOVA) with repeated measures was applied to examine differences between the time points (baseline, pre-match, half-time, and post-match) and groups (experimental and control), followed by Tukey’s post hoc test if required. In addition, an unpaired t-test was also used to identify significant differences in TRIMP between the match halves and the groups’ anthropometric characteristics. All data analyses were performed using the Statistica software (version 12.0, StatSoft Inc., Tulsa, OK, USA), and a significance level of *p* ≤ 0.05 was chosen. GraphPad Prism (version 10.0, GraphPad Software Inc., San Diego, CA, USA) was used to create the graphs presented in this study.

## 3. Results

**Figure 1:**
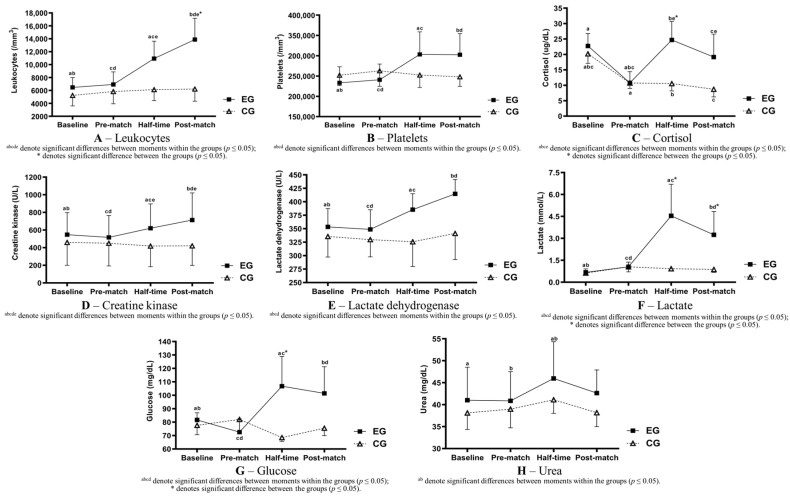
Blood markers’ behavior following the football match (mean ± SD). ^abcde^ denote significant differences between moments within the groups (*p* ≤ 0.05); * denotes significant differences between the groups (*p* ≤ 0.05).

The results of the TRIMP show higher demands for the first half compared with the second half of the match (1T: 180.8 ± 13.0; 2T: 168.0 ± 16.1; *p* = 0.020). Figure 1 shows the blood markers’ behavior following the experiment.

Considering the groups of blood markers analyzed, the immunological blood marker group was the one showing higher effects following the match, as displayed in Table 3.

The total leukocyte count was significantly higher between moments and groups (EG baseline: 6470 ± 1541; EG post-match: 13,890 ± 3292; Figure 1A). Platelets showed similar behavior, with significant differences between moments (EG baseline: 233,300 ± 39,497; EG post-match: 302,400 ± 52,073; Figure 1B); however, no differences between the groups were found. Cortisol showed a significant increase for the half-time and post-match stages, compared with the pre-match stage, for the EG, even though the measurements for the post-match stage were significantly lower than the half-time stage (pre-match: 10.78 ± 3.63; half-time 24.70 ± 6.04; post-match: 19.15 ± 7.40; Figure 1C). Between the groups, the only moment with differences compared with the CG was at half-time.

As shown in Table 4, a significant increase was found in the EG in-between moments for CK (baseline: 547.20 ± 250.74; post-match: 713.70 ± 308.20; Figure 1D) and LDH (Baseline: 353.40 ± 34.08; post-match: 414.80 ± 26.55; Figure 1E). No differences between the groups were identified.

Amongst the metabolic markers, as per Table 5, similar results were found for lactate and glucose: the EG’s values were significantly higher during the half-time and post-match stages compared with the pre-match stage (lactate, baseline: 0.67 ± 0.24; half-time: 4.54 ± 2.16; post-match: 3.24 ± 1.60; Figure 1F; glucose, baseline: 81.65 ± 5.29; half-time: 106.79 ± 22.02; post-match: 101.42 ± 19.87; Figure 1G). When comparing the groups, the EG’s lactate was significantly higher during the half-time and post-match stages, whereas the EG’s glucose increased at half-time only. A significant increase for the EG when comparing the half-time and pre-match values was also found for urea (pre-match: 40.87 ± 6.65; half-time: 45.96 ± 8.38; post-match: 42.64 ± 5.26; Figure 1H). No significant alterations were found between moments and groups for the other metabolic markers such as erythrocytes, hematocrit, and hemoglobin.

Table 6 displays a significant increase only between the half-time and pre-match stages between the groups for potassium (pre-match: 7.29 ± 0.98; half-time: 5.79 ± 0.89; post-match: 6.87 ± 1.00) and sodium (pre-match: 136.40 ± 1.07; half-time: 138.00 ± 1.49; post-match: 136.80 ± 1.48). No remarkable differences were found for the other markers analyzed.

## 4. Discussion

The present study investigated the acute effects of a football match on immunological, muscle damage, metabolic, and electrolytic blood markers by comparing four time points between the EG and CG. Consistent with our hypothesis, the football match generated a noteworthy response in immunological markers, with regard to leukocytes and cortisol, which presented significant differences between moments and groups. Likewise, CK and LDH increased significantly across the moments for the EG; however, no difference between the groups and raised lactate and glucose between the groups at half-time were also observed. In contrast with our hypothesis, the electrolytic markers did not show any significant response when comparing EG and CG, emphasizing the importance of including a CG in the study design. In addition, as shown by the TRIMP analysis, the first-half physical demands were higher compared with the second half, which is in line with findings from previous research [37,38,39].

Immunological-related blood markers showed higher effects following the experimental activity, especially the participants’ white cell count (leukocytes), which was significantly higher compared with the pre-match stage during the half-time and post-match stages. When comparing the groups, statistical differences were also found during the post-match stage, even though no differences were found between moments for the CG. These findings are in line with other studies with similar methodological designs, highlighting that playing a football match might generate inflammatory responses in the body’s defense system, stimulating leukocyte infiltration to promote tissue regeneration [17,19,24]. Equivalent results were found for platelets, whose primary function is to help the blood to clot; however, no differences between the groups were identified. Similar results have been found in other studies and the possible physiological underpinning is the increment of platelet clearance due to spleen activity, therefore increasing the number of platelets in the blood [40,41].

Regarding cortisol, our findings are in line with other studies [17,18], although the half-time analysis was not included in any of them, thereby impeding the analysis of the reduction observed in the post-match stage in comparison with the half-time stage in the EG. Nonetheless, this may be explained by the higher demands during the first half, as cortisol has catabolic functions, such as increased protein degradation and decreased protein synthesis in peripheral tissues [42]. In addition, our findings could be associated with the facilitator role of cortisol in picking substrates, as this will be expressed in the results for glucose, lactate, and urea thereafter.

Concerning muscle damage indicators, CK and LDH are enzymes located in the skeletal muscle cells; they take part in the alactic and lactic anaerobic systems, respectively, and are released to the blood serum in response to muscle micro-ruptures [43,44]. In this investigation, CK and LDH levels progressively increased during the half-time and post-match stages, compared with the pre-match values, but no differences between the groups were found. These findings are in agreement with other studies with a CG, supporting the idea that football matches elicit acute muscle micro-damage and inflammation [17,18,19,20]. High-intensity acceleration and deceleration efforts are important measures of external load in team sports [45] and have been considered the largest contributors to post-match muscle damage blood markers regarding CK [46]. Indeed, these types of activities require a considerable amount of concentric and especially eccentric muscle activation, which in turn elicits higher cross-sectional tension, therefore resulting in significant muscle damage [23,47,48]. High-intensity running is also associated with changes in CK, as, for every 100 m above 5.5 m/s, post-match CK may increase by approximately 30% [49].

Amongst the metabolic indicators, analogous results for lactate and glucose were found, as both markers increased during the half-time and post-match stages, compared with the pre-match stage. Although there have been no studies reporting a CG in association with half-time analysis for these indicators, the current findings are similar to those of previous studies [31,50]. Increases in serum glucose and lactate support the fact of a considerable anaerobic metabolism contribution during football matches in order to quickly cope with the demands of high-intensity activities [31,50]. A higher lactate production may not represent an effect of a single action; however, it is linked to an accumulated response to several high-intensity activities [31]. In addition, the post-match reduction in comparison to the half-time values can also be explained by the lower second-half physical demands, which was previously demonstrated by the TRIMP analysis. Moreover, a progressive decrease in insulin and elevation in catecholamine levels throughout a football match have also been described [50], which stimulate a higher rate of lipolysis [16,36]. This process may be associated with lowered post-match lactate levels compared to half-time, indicating increased fat oxidation in compensation for the lower levels of muscle glycogen [51]. A raised lactate concentration can also be explained by high-intensity activities performed immediately before the blood collection [52]. Concerning urea, the values for the half-time stage were significantly higher compared to the pre-match stage. In contrast to previous studies [18,53], no difference was found between post- and pre-match moments, which may be explained by the lower second-half internal demands.

Playing a football match might also induce changes in electrolytic markers as the game leads to elevations in body temperature, and consequently, sweating responses are initiated to promote heat loss [54]. In the present study, sodium presented higher values for the half-time stage when comparing both groups; however, despite this discrepancy, for all samples, the values were within the expected reference [55], between 135 and 145 mEq/L. An analogous result was found for potassium (7.53 ± 0.82 to 5.79 ± 0.89 mEq/L). Moreover, an increment in calcium at half-time and a decrement in the post-match stage for both groups were observed, reinforcing the importance of considering that a CG as the outcome might be related to the circadian rhythm [56]. Water intake may also affect changes in electrolytic markers (Table 2).

A potential limitation of the present study was that the external load demands could not be assessed during the experimental protocol; only the internal load was assessed. Moreover, as the association between blood markers and TRIMP was not investigated, the main factors that may influence physical performance during football matches were not evaluated. Body composition, regarding lean body mass, was also not associated with the blood marker changes. Finally, the limited number of participants included might also harm the generalization of the present findings. Future research should attempt to identify blood markers’ match-to-match variability, as well as the association between external match demands and the acute response of internal indicators.

## 5. Conclusions

A football match at a high-performance level provokes acute disturbances in immunological, muscle damage, and metabolic blood markers, with no significant alterations in the electrolytic indicators. Moreover, as shown in our current study, considering the fact that including a CG associated with half-time analysis added remarkable value, despite some markers expressing an increase between match moments for the EG, no significant differences were found when comparing this group with the CG.

According to the inflammatory and muscle damage responses found in the present study, practitioners should tailor post-match recovery strategies so that the players can perform at the highest level in an abbreviated period. In addition, post-match fueling strategies are considerably important to quickly restore the muscle glycogen consumed during the match. Finally, particular metabolic and electrolytic blood makers should be carefully used as exertion indicators, as no significant changes were found for erythrocytes, hematocrit, hemoglobin, sodium, calcium, and potassium following a football match.

## Figures and Tables

**Table 1:** Subjects’ anthropometric and cardiorespiratory characteristics (mean ± SD).

	Experimental Group	Control Group	*p*-Value
Age (y/o)	18.33 ± 0.44	17.91 ± 0.53	0.1145
Height (cm)	177.35 ± 4.15	182.58 ± 8.40	0.1147
Body weight (kg)	75.32 ± 3.25	80.85 ± 5.73	0.0260 *
%Bodyfat	11.20 ± 1.10	11.90 ± 1.46	0.2945
HRrest (bpm)	68.2 ± 11.4	59.8 ± 3.9	0.1804
HRmax (bpm)	198.1 ± 8.2	195.0 ± 7.4	0.5251
VO2max (mL/kg/min)	57.22 ± 3.53	54.92 ± 9.35	0.5010

* denotes significant differences between the groups (*p* ≤ 0.05).

**Table 2:** Water intake registry (mean ± SD).

	Experimental Group	Control Group
Pre-match (mL)	312.5 ± 200.8	336.0 ± 143.7
Half-time (mL)	123.3 ± 67.7	439.5 ± 107.7 *

* denotes significant differences between the groups (*p* ≤ 0.05).

**Table 3:** Immunological blood markers’ behavior (mean ± SD).

Marker	Group	Baseline	Pre-Match	Half-Time	Post-Match
Leukocytes	EG	6470 ± 1541 ^ab^	6920 ± 1949 ^cd^	10,940 ± 2683 ^ace^	13,890 ± 3292 ^bde^
(/mm^3^)	CG	5233 ± 1623	5833 ± 1912	6133 ± 1710	6217 ± 1895
Platelets	EG	233,300 ± 39,497 ^ab^	240,700 ± 38,807 ^cd^	303,300 ± 55,627 ^ac^	302,400 ± 52,073 ^bd^
(/mm^3^)	CG	252,000 ± 25,479	262,833 ± 38,426	252,333 ± 30,480	248,000 ± 23,698
Cortisol	EG	22.73 ± 4.06 ^a^	10.78 ± 3.63 ^abc^	24.70 ± 6.04 ^bd^*	19.15 ± 7.40 ^cd^
(µg/dL)	CG	20.20 ± 3.10 ^abc^	10.75 ± 1.77 ^a^	10.55 ± 2.37 ^b^	8.70 ± 2.47 ^c^

^abcde^ denote significant differences between moments within the groups (*p* ≤ 0.05); * denotes significant difference between the groups (*p* ≤ 0.05).

**Table 4:** Muscle damage blood markers’ behavior (mean ± SD).

Marker	Group	Baseline	Pre-Match	Half-Time	Post-Match
CK	EG	547.20 ± 250.74 ^ab^	516.50 ± 248.38 ^cd^	620.30 ± 276.12 ^ace^	713.70 ± 308.20 ^bde^
(U/L)	CG	459.50 ± 260.47	450.50 ± 258.44	418.50 ± 234.56	420.67 ± 222.59
LDH	EG	353.40 ± 34.08 ^ab^	348.80 ± 36.49 ^cd^	385.40 ± 29.62 ^ac^	414.80 ± 26.55 ^bd^
(U/L)	CG	335.50 ± 38.11	329.83 ± 32.16	325.83 ± 45.85	341.17 ± 48.35

^abcde^ denote significant differences between moments within the groups (*p* ≤ 0.05).

**Table 5:** Metabolic blood markers’ behavior (mean ± SD).

Marker	Group	Baseline	Pre-Match	Half-Time	Post-Match
Lactate	EG	0.67 ± 0.24 ^ab^	1.05 ± 0.32 ^cd^	4.54 ± 2.16 ^ac^*	3.24 ± 1.60 ^bd^*
(mmol/L)	CG	0.63 ± 0.17	1.05 ± 0.34	0.92 ± 0.12	0.87 ± 0.20
Glucose	EG	81.65 ± 5.29 ^ab^	72.54 ± 9.76 ^cd^	106.79 ± 22.02 ^ac^*	101.42 ± 19.87 ^bd^
(mg/dL)	CG	77.65 ± 7.01	82.03 ± 10.46	68.48 ± 2.82	75.53 ± 5.64
Erythrocytes	EG	5.21 ± 0.26 ^ab^	5.03 ± 0.19 ^a^	5.11 ± 0.21	5.06 ± 0.25 ^b^
(millions/mm^3^)	CG	5.06 ± 0.30 ^a^	4.94 ± 0.29	4.88 ± 0.24	4.82 ± 0.17 ^a^
Hemoglobin	EG	14.72 ± 1.00 ^a^	14.20 ± 0.94 ^a^	14.51 ± 1.03	14.43 ± 0.89
(g/dL)	CG	14.78 ± 1.09 ^a^	14.40 ± 0.81	14.32 ± 0.67	14.25 ± 0.80 ^a^
Hematocrit	EG	44.56 ± 2.30 ^ab^	42.81 ± 2.09 ^a^	43.54 ± 2.20	42.88 ± 1.88 ^b^
(%)	CG	44.37 ± 3.14 ^a^	43.37 ± 2.36	42.75 ± 2.44	42.43 ± 1.91 ^a^
Urea	EG	41.02 ± 7.48 ^a^	40.87 ± 6.65 ^b^	45.96 ± 8.38 ^ab^	42.64 ± 5.26
(mg/dL)	CG	38.12 ± 3.78	38.98 ± 4.27	41.12 ± 3.11	38.17 ± 3.18

^abcd^ denote significant differences between moments within the groups (*p* ≤ 0.05); * denotes significant differences between the groups (*p* ≤ 0.05).

**Table 6:** Electrolytic blood markers’ behavior (mean ± SD).

Marker	Group	Baseline	Pre-Match	Half-Time	Post-Match
Calcium	EG	9.83 ± 0.26 ^ab^	10.33 ± 0.28 ^ac^	10.12 ± 0.33 ^d^	9.37 ± 0.29 ^bcd^
(mg/dL)	CG	9.44 ± 0.34 ^abc^	10.06 ± 0.13 ^ad^	9.97 ± 0.37 ^be^	8.89 ± 0.32 ^cde^
Sodium	EG	138.3 ± 1.06 ^a^	136.4 ± 1.07 ^ab^	138.0 ± 1.49 ^b^*	136.8 ± 1.48
(mEq/L)	CG	138.0 ± 0.89 ^ab^	136.0 ± 0.89 ^a^	135.3 ± 0.82 ^b^	136.3 ± 0.82
Potassium	EG	3.75 ± 0.20 ^abc^	7.29 ± 0.98 ^ad^	5.79 ± 0.89 ^bde^*	6.87 ± 1.00 ^ce^
(mEq/L)	CG	3.97 ± 0.12 ^abc^	6.95 ± 0.76 ^a^	7.53 ± 0.82 ^b^	6.67 ± 0.65 ^c^

^abcde^ denote significant differences between moments within the groups (*p* ≤ 0.05); * denotes significant differences between the groups (*p* ≤ 0.05).

## Data Availability

The data presented in this study are available from the corresponding author upon request. The data are not publicly available due to privacy restrictions.
